# Blunt Thoracic Aortic Injury

**DOI:** 10.3390/jcm12082903

**Published:** 2023-04-17

**Authors:** Daniela Mazzaccaro, Paolo Righini, Fabiana Fancoli, Matteo Giannetta, Alfredo Modafferi, Giovanni Malacrida, Giovanni Nano

**Affiliations:** 1 Operative Unit of Vascular Surgery, IRCCS Policlinico San Donato, San Donato Milanese, 20097 Milan, Italy; 2 Department of Biomedical Sciences for Health, University of Milan, 20122 Milan, Italy

**Keywords:** aortic, thoracic, trauma, blunt injury, endovascular

## Abstract

Blunt thoracic aortic injury (BTAI) is a potentially fatal condition that needs prompt recognition and expedited management. Clinical manifestations of BTAI are not straight forwarding and may be misdiagnosed. The grade of aortic injury is an important determinant of perioperative mortality and morbidity, as well as the indication of treatment, along with the presence of concomitant lesions of other involved organs. The mainstay of treatment nowadays for hemodynamically stable patients who survive the trauma scene is represented by delayed endovascular repair whenever anatomically and clinically feasible. Endovascular repair, in fact, is burdened by lower perioperative mortality and morbidity rates if compared to open surgical repair, but concerns remain about the need for long-term surveillance and radiation exposure in patients who are at a younger age than patients treated for the aneurysmal disease. The aim of the paper is to provide an update on the diagnostic modalities and strategies of treatment for patients affected by BTAI.

## 1. Introduction

Blunt thoracic aortic injury (BTAI) may occur in patients who sustain thoracic trauma with rapid deceleration, either from high-speed motor vehicle collision or falls from a significant height [1]. The aortic isthmus, just distal to the left subclavian artery, is the most involved site [2], but other sites also can be affected.

The reason why the aortic isthmus is the most affected zone lies in the relative weakness of this segment of transition between the more mobile ascending aorta and arch and the relatively fixed descending thoracic aorta, which is furthermore trapped between anterior and posterior bony structures, leading to focal rupture [3].

From a histologic point of view, the aortic rupture occurs first as intimal and medial injury, followed by the adventitial rupture that can occur during an unpredictable interval of time ranging from seconds to years [4]. If the rupture is immediate, the patient dies directly on the trauma scene.

BTAI is then a serious condition that can rapidly lead to death, but the clinical presentation features sometimes lack sensitivity and specificity and pose challenges to physicians dealing with such situations. Prompt diagnostic evaluation, often with a multidisciplinary approach, as well as appropriate treatment, is mandatory to reduce mortality and morbidity rates in those who are able to arrive at the hospital.

The aim of this paper is then to provide a narrative review of the literature about the most frequent clinical presentations of BTAI and to provide an update on the diagnostic modalities and strategies of treatment for patients affected by BTAI.

## 2. Clinical Features of BTAI

The trauma patient who can present a BTAI may arrive at the Emergency Department (ED) with a complaint of chest pain, interscapular pain, or difficulty breathing. One-third of the patients present with hypotension, and about 40% of them may present with an altered state of consciousness [5].

Nevertheless, the patient may present with lethal lesions and may not survive after immediate arrival at the ED. Dinh et al. [1], in their single-center experience, described that about 17% of patients who arrived with a BTAI died in the ED.

Patients who are victims of BTAI are predominantly young or mid-aged males since the most represented mechanism of injury is usually road traffic accidents [6].

The history of thoracic trauma should always be investigated in patients who arrive in ED after a motor vehicle collision or a fall. In particular, the steering wheel or seatbelt imprint on the surface of the skin should suggest a thoracic trauma with a rapid deceleration. Left subclavicular hematoma and a previously unknown cardiac murmur also can be associated with BTAI but are not typical.

On physical examination, patients may also present bilateral hyposphygmia of femoral pulses with increased pressure of the upper extremities. Arrhythmia, low blood pressure, tachycardia, and signs of peripheral vascular shutdown may suggest the presence of severe hypotensive status.

The trauma patient may also present other associated injuries with respect to the different mechanisms of damage. Commonly associated injuries include head, lung, and heart contusions, intra-abdominal hemorrhage, bone fractures (usually pelvic and spinal), and diaphragmatic rupture [7,8]. Scalea et al. [5] reviewed the data of the National Trauma Databank (about 3774 patients over 11 years), and they found that the most frequently associated traumatic injuries were those to the lower extremities (36%), the head (32.5%), and the abdomen (28%). The presence of associated injuries may increase the hemodynamic instability of the patients, which in turn may increase the mortality risk, irrespectively from the presence of an aortic injury [6]. Bade-Boon et al., in their review, found that patients who arrive with BTAI may have an Injury Severity Score that ranges from 31 to 54 [6].

Laboratory tests are not specific for BTAI, but they can reflect the general severity of the injury; for example, the presence of acute blood loss or the presence of acute kidney injury.

At plain anteroposterior chest X-ray, indirect signs can be noted, such as a wide mediastinum, abnormal aortic arch imprint, deviation of the trachea, and bone fractures.

All of them, however, are not specific for BTAI; therefore, further imaging testing is required. Crapps et al. analyzed the results of 708 patients with confirmed BTAI injuries, and they described that the most consistent single finding identified at plan chest X-ray was widened mediastinum, but this finding was present in 27.7% of all confirmed BTAIs and in about half of the patients who presented with a high-grade injury [9].

## 3. Diagnosis

Early diagnosis of BTAI is crucial to reduce mortality rates, particularly in severely diseased patients. Boutin et al., in fact, in their multicenter retrospective study, found that the time to diagnosis increased with the severity of the aortic injury and the clinical severity of the patients [10].

While a whole-body multidetector computed tomographic scan (CT) is often performed in the acute management of trauma patients [11] when suspecting a BTAI, the mainstay imaging modalities are represented by contrast-enhanced CT (CE-CT) of the chest and transesophageal echocardiography (TEE) [12]. There are concerns about radiation exposure when performing a whole-body CE-CT.

In particular, CE-CT is recommended for the diagnosis of BTAI in hemodynamically stable patients, while TEE is a valuable alternative for hemodynamically unstable patients who require prompt bedside assessment. On the other side, transthoracic echocardiography is not indicated for BTAI [13].

**Figure 1:**
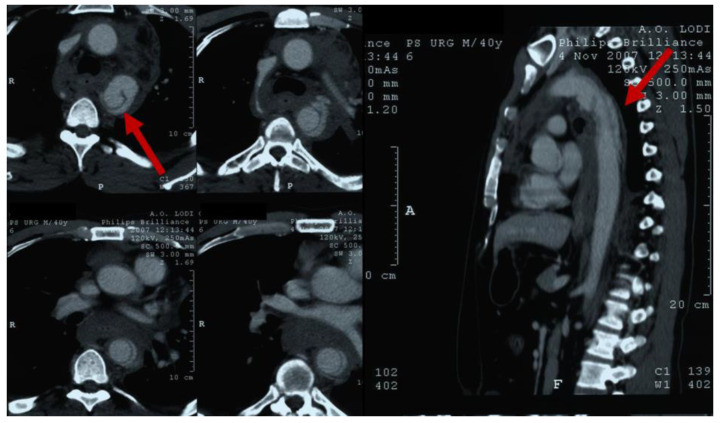
Angio-Computed Tomography chest scan of a patient with BTAI at isthmus, causing intimal tear (red arrow) in axial (on the **left**) and sagittal (on the **right**) views.

Both CE-CT and TEE can identify early sub adventitial aortic injuries with comparable diagnostic accuracies. CE-CT of the chest, in particular, is a highly sensitive and specific test for BTAI, having a reported sensitivity of 95–100% and a negative predictive value of 100% [14]. It can be useful also to identify other associated lesions, such as lung contusions. Furthermore, chest CE-CT investigates the aortic segment from the ascending to the thoraco-abdominal district, with the possibility of extending the evaluation also to the abdominal region. Findings on chest CE-CT indicative of BTAI are the presence of intimal damages, from flap (Figure 1) to complete vessel wall disruption with contained or total rupture, the presence of luminal filling defects, and the presence of aortic contour abnormalities such as periaortic hematoma or pseudoaneurysm.

TEE can provide a similar evaluation in hemodynamically unstable patients who cannot be moved safely from the Emergency Department or from the Operating Room [15]. In the hands of an experienced echocardiographer, TEE can have a sensitivity that ranges between 91% and 100% and a huge specificity ranging from 98% to 100%, especially for the evaluation of the ascending aorta and the isthmus [16].

**Figure 2:**
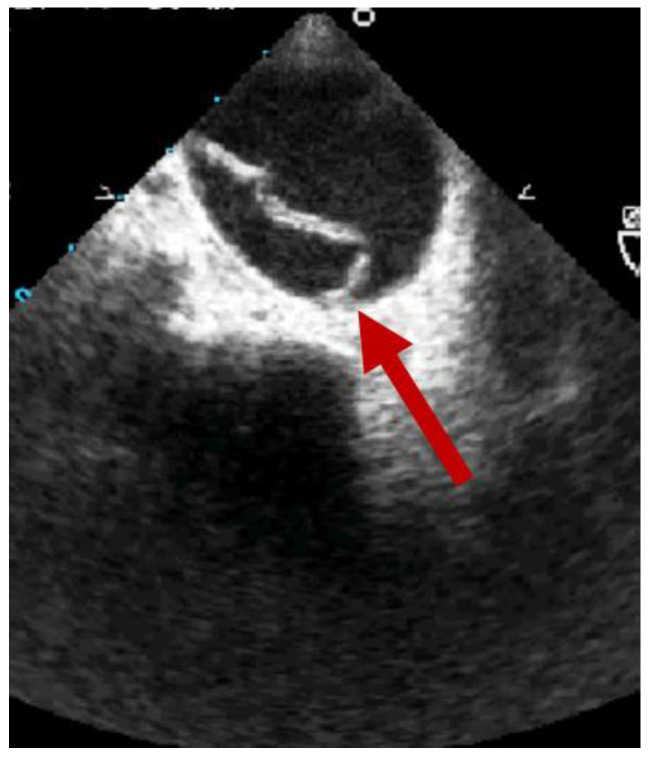
Transesophageal echocardiography showing the presence of an intimal flap (red arrow).

At TEE, an irregular intraluminal flap can be seen traversing the lumen of the aortic isthmus in the transverse view (Figure 2), as well as the presence of an abnormal aortic contour suggesting an acute pseudoaneurysm [15]. The addition of the color Doppler and the continuous wave Doppler evaluations can help in identifying local blood flow turbulence that can suggest wall disruption [15] or intermittent aortic obstruction with pseudo-coarctation syndrome.

The presence of a mediastinal hematoma can also be accurately diagnosed using TEE [15], as well as the presence of wall thrombi within the aortic lumen, which are typically located in the proximal descending thoracic aorta.

Nevertheless, the guidelines strongly recommend CE-CT to diagnose BTAI because of its availability, rapidity, and ability to diagnose additional intra-thoracic injuries [12].

Thoracic aortography is not used for the diagnosis of BTAI, given the greater invasiveness and the lower sensitivity in comparison to CE-CT [17]. Magnetic resonance (MR) imaging has similar results to CE-CT, but it is uncommonly used for BTAI due to the time needed for image acquisition.

Intravascular ultrasound (IVUS) can be a valuable tool when used at the time of endovascular repair of stable patients since it facilitates accurate endograft sizing and graft deployment [18].

## 4. Aortic Injury Grading

**Figure 3:**
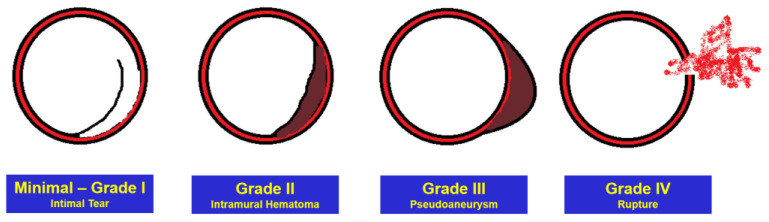
Grade of aortic injuries.

Several classification schemes for grading the severity of aortic injury have been proposed [19], but the most used is a relatively simple system on which indications and recommendations for treatment are based [20], Figure 3:

- Grade I: Intimal tear.
- Grade II: Intramural hematoma.
- Grade III: Pseudoaneurysm.
- Grade IV: Rupture.

In addition, the term “minimal aortic injury” has been used to describe relatively small lesions, such as isolated intimal defects less than 1 cm without periaortic mediastinal hematoma [21]. While small intimal tear (Grade I) has always been considered a minimal aortic injury, the relatively recent inclusion of sub-centimetric intramural hematoma (Grade II) is strongly supported by newer management-based classification systems (e.g., the Vancouver and Harborview classifications) [21]. It is characterized by increased attenuation within the aortic wall due to hemorrhage from ruptured vasa vasorum or small intimomedial tears and represents less than 5% of “minimal aortic injuries” [21]. According to Malhotra et al. [22], approximately 10% of patients with BTAI have “minimal aortic injury” at CT angiography.

## 5. Initial Management

The clinical presentation and the hemodynamic status of the patient, together with the grade of aortic injury, are all main determinants of the indication of treatment, as well as of the timing of treatment [13].

More specifically, injury grade is a predictor of aortic-related mortality among patients with BTAIs [20], in particular, high-risk patients with Grade III and Grade IV injuries.

As a general rule, patients with BTAIs should be treated at a trauma center that has experienced and expert professionals and opportune facilities for perioperative care. If this level of care is not available at the department to which the patient has come, the patient should be stabilized and transferred to a more suitable facility.

The common principles of general trauma management include a careful evaluation of the vital signs for the prompt identification of life-threatening injuries, according to the basic rules of Advanced Trauma Life Support [23].

The trauma team should include the general surgeon, the emergency physician, the orthopedic surgeon and the critical care/anesthesia specialist on call, and at least two trained nurses and two paramedics. Patent with cervical spine precautions should be established, followed by the administration of supplemental oxygen as appropriate, with a target of achieving 94–98% saturation. If tension pneumothorax is detected, immediate drainage should be performed to relieve the tension [24]. Then, hemorrhage control should be achieved, and fluid resuscitation should be started after placement of one or more robust intravenous accesses if systolic blood pressure is less than 90 mmHg or heart rate is greater than 120. In case of hemorrhagic shock, calcium chloride is also suggested, 20 mg/kg (0.2 mL/kg), max of 1000 mg (10 mL). Clinical signs of traumatic brain injury should then be assessed, as well as the presence of other concomitant injuries. It is important to avoid hypothermia, remove wet clothing, and cover the patient to prevent further heat loss [24].

### 5.1. Minimal Aortic Injuries

“Minimal” injuries can be managed conservatively with safe results [21].

In such cases, the approach consists primarily of antihypertensive therapy, which should be initiated on admission with intravenous beta blockers or negative inotropic drugs (such as esmolol) to achieve a systolic blood pressure lower than 100 mmHg and a heart rate < 100 beats per minute. This approach has the main goal of reducing the risk of extending the injury and reducing the volume of blood loss in case of aortic rupture. Some studies have observed a reduction in mortality in patients treated with antihypertensive therapy [25] compared to untreated patients.

In patients in whom beta blockers are contraindicated, a calcium channel blocker, such as diltiazem, can be used. If beta-blocker therapy is not enough to control systolic blood pressure, a vasodilator such as nitroprusside can be used in combination. There is no consensus about the optimal duration of antihypertensive therapy.

Nevertheless, the target for systolic blood pressure in case of concomitant head injury is 110 mmHg. Hypotension should, in fact, be avoided to maintain cerebral perfusion [24].

Then, the patient should undergo serial imaging during follow-up, mainly using a chest CE-CT scan [26]. However, there is no consensus about the optimal timing for the imaging, which is left to the practitioner based on each clinical case.

### 5.2. Grade II, III, and IV Aortic Injuries

#### 5.2.1. Hemodynamically Unstable Patients

Based upon the principles of Advanced Trauma Life Support (ATLS), hemodynamically unstable trauma patients (such as Grade IV aortic injuries) should undergo emergent surgical exploration to determine and control the source of life-threatening hemorrhage. In the case of thoracic aortic injury, the use of the resuscitative endovascular balloon occlusion of the aorta (REBOA) is contraindicated.

#### 5.2.2. Hemodynamically Stable Patients

According to major trauma association guidelines [13], hemodynamically stable patients with type II and III and no signs of impending thoracic aortic rupture, significant aortic thrombus, massive pneumothorax, or luminal encroachment can be treated with delated aortic repair, after aggressive heart rate and blood pressure control [20,27], especially if the patient has severe coexistent injuries. Immediate aortic repair, in fact, in such cases, may not be feasible or even associated with worse outcomes and a higher risk of postoperative death and paraplegia [13].

High-risk patients with favorable anatomy may better benefit from endovascular rather than open repair [13,19].

In such cases also, delayed repair should be the choice whenever feasible. Romijn et al., in fact, in their propensity-score analysis of 548 matched patients submitted to Thoracic Endovascular Aortic Repair (TEVAR) for BTAI, found that immediate (<24 h) repair was associated with a two-fold higher mortality if compared to delayed repair (>24 h), even after adjusting for the injury grade [27].

## 6. Aortic Repair

Over the past 30 years, the preferred approach for the treatment of thoracic aortic diseases has moved from open repair to the less invasive use of endovascular stent grafts [12].

This concept also applies to the treatment of BTAIs, where the preference is toward endovascular repair whenever possible [5]. Open surgical repair, however, still has a role when the anatomic features preclude endovascular repair or when there is the need for open thoracic surgery to treat other associated injuries.

### 6.1. Endovascular Repair

The endovascular procedure for the treatment of BTAIs is similar to what is performed for other aortic thoracic injuries, with reported technical success rates ranging from 80 to 100% [28].

Similar to what happens for the endovascular treatment of aortic dissections or thoracic aneurysms, the placement of the endovascular graft can be aided by the use of intravascular ultrasound and/or transesophageal echocardiography [29].

In particular, the use of trans-esophageal echocardiography (TOE) may be considered for the guidance of wire placement via the dissected aorta, for endoleak assessment, and/or for the detection of cannulation injury. The benefit of TOE in these scenarios is well supported by evidence [30].

Furthermore, similar to the treatment of aneurysmal disease, spinal fluid drainage may be used to prevent spinal cord ischemia in case of long (>20 cm) endograft coverage, if there has been a previous abdominal aortic repair, or in case area of possible coverage of the artery of Adamkievicz.

Nevertheless, the endovascular treatment of BTAIs deserves particular considerations that mainly concern the sizing of the endovascular graft and the management of intraoperative anticoagulation.

As for the first issue, it is worth keeping in mind that thoracic stent graft devices were originally designed to treat aneurysmal diseases, and about 20 to 30% of them are not suited to the narrow and tight angulation of the aortic arch, which is typical of the younger patients who present with BTAI [31]. Furthermore, the size of the aorta in patients affected by BTAI may be underestimated by the low systolic pressure, especially in hemodynamically unstable patients. This may, in turn, lead to an underestimation of the graft sizing with a consequent inappropriate degree of oversizing to ensure proximal fixation [32] with device malapposition [33], infolding, or compression [34]. Nevertheless, some authors suggest being cautious with the oversizing of the endograft in such cases and that 10% oversizing may be appropriate [35].

Neschis et al., in their experience, about 43 patients aged in mean 44 years treated for BTAI, reported an average aortic diameter of about 23 mm, ranging from 19 to 30 mm [36].

**Figure 4:**
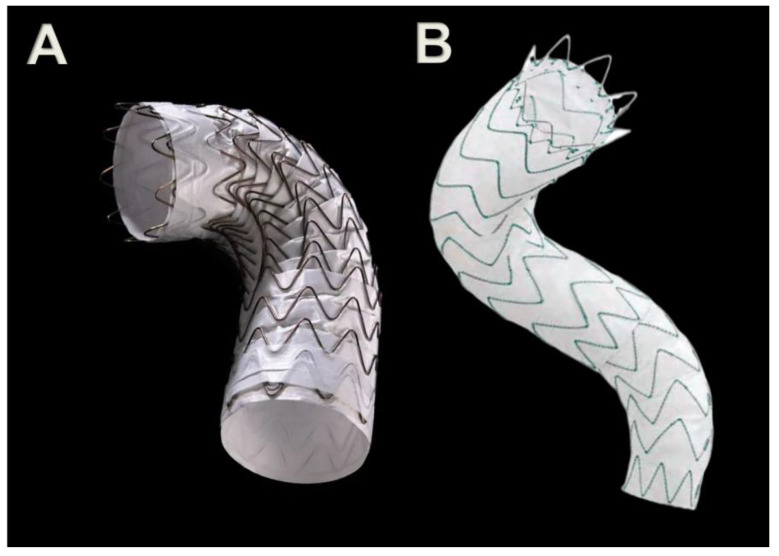
The Conformable GORE TAG^®^ (C-TAG) thoracic stent graft (W. L. Gore & Associates, Flagstaff, AZ, USA) on the left side (**A**), and the Medtronic Valiant Captivia™ (Medtronic Inc., Santa Rosa, CA, USA) on the right side (**B**).

Up to now, no devices are available for aortic diameters lower than 16 mm, and there are actually only two devices, among the endograft used for standard thoracic endovascular aortic repair, that have received approval for use also in BTAI. Both of them cover similar indications: the Conformable GORE TAG^®^ (CTAG) thoracic stent graft (W. L. Gore & Associates, Flagstaff, AZ, USA), and the Medtronic Valiant Captivia™ (Medtronic Inc., Santa Rosa, CA, USA) (Figure 4, Table 1).

To ensure an optimal proximal landing zone, coverage of the left subclavian artery may be required, particularly when the injury is located at the level of the aortic isthmus.

In such cases, the revascularization of the subclavian artery using an extra-anatomic bypass should be performed to reduce the risk of neurologic complications [37]. In fact, while prior studies have suggested that the left subclavian artery could be covered relatively safely in BTAI, preliminary multicenter prospective data suggest a significant increase in ischemic events without revascularization of the left subclavian artery [38].

Some peculiar cases, such as the presence of a “bovine” aortic arch, are associated with a consistent geometric pattern, which identifies hostile proximal landing zones for endograft deployment, namely Zone 3 and Zone 0 in the Type I arch. This mandates a specific amendment to thoracic endovascular aortic repair planning, requiring, for example, the need for a total debranching of the supra-aortic trunks to ensure a safer proximal landing zone [39] or the use of custom-made branched/fenestrated devices.

The second issue concerns the indication for anticoagulation. In particular, the administration of unfractionated heparin (UFH) during endovascular repair of BTAI is controversial, and patients with traumatic aortic injury may present significant associated injuries, such as intracranial hemorrhage or splenic injury, that may contraindicate anticoagulation. In such circumstances, an endovascular repair can be performed without the use of heparin, without significant adverse consequences [40].

Nevertheless, Makaloski et al., in their retrospective analysis of 36 patients submitted to TEVAR for BTAI, found that, in patients with hemodynamic stability and no severe associated injuries, systemic anticoagulation can be safely performed with no intraoperative bleeding or thromboembolic complications in the early postoperative period [41].

### 6.2. Open Surgical Repair

Open surgical repair of BTAI is similar to open repair of thoracic aortic aneurysm, keeping, however, in mind some peculiarities.

The first concerns the site of the incision, which is based on the location of the pathology and the need to obtain a safe proximal aortic cross-clamping control. Isolated BTAIs occurring at the aortic isthmus require, for example, a thoracotomy incision in the fourth intercostal space and aortic cross-clamping just distal to the left subclavian artery or between the left common carotid artery and the left subclavian artery.

Then, active perfusion of the distal aorta, through either left atrial-femoral bypass or femoral venous-to-femoral arterial cardiopulmonary bypass, may be performed to reduce the incidence of perioperative paraplegia [42], even in case of short cross-clamping times (<30 min). Femoral venous–arterial bypass may have more advantages compared to the former technique, such as the possibility of simultaneous cooling of the patient, which may augment spinal cord protection, the reduced need for systemic heparin administration, and the possibility to oxygenate the blood independently of the lung, which may be worthy particularly in patients with concomitant lung injuries [43].

Open surgical repair of the aorta may also be preliminary to a secondary endovascular intervention, such as when treating complex lesions involving the aortic arch and the descending thoracic aorta. In such cases, the “Frozen Elephant Trunk” (FET) technique may be used to perform the two-staged approach to repair the complex lesion. New branched hybrid grafts, such as the Thoraflex, the Cronus (MicroPort, Shanghai, China), the Evita Open Neo (Atrivion Inc., Kennesaw, GA, USA), and the J graft (now Frozenix) (Japan Lifeline, Tokyo, Japan), are currently available on the market. They enable the reimplantation of the supra-aortic vessels separately using prefabricated vascular branches, and a side graft allows direct cannulation for antegrade distal perfusion during arch replacement [30].

Furthermore, hybrid surgery combining rapid resuscitative thoracotomy, direct suturing of the aortic lesion, and completion of endovascular treatment may be used as a safe treatment strategy in complex cases [44].

## 7. Perioperative Outcomes

The introduction of endovascular techniques over open repair for the treatment of BTAIs has dramatically improved perioperative outcomes [45]. The reported perioperative mortality rates after endovascular repair range about 7–9% [46], while it is about 19% in patients treated with open surgery [47].

Irrespective of the chosen technique, the main reported perioperative complications to include spinal cord ischemia and stroke.

Spinal cord ischemia, which may result in paraparesis or paraplegia, remains a significant concern, particularly after open repair, where it has been reported in up to 9% percent of cases [48]. On the other hand, Murad et al., in their systematic review, reported a 3% risk of spinal cord ischemia after endovascular repair of BTAIs [47].

Perioperative stroke is another potentially devastating complication, especially if the aortic injury is proximal to the origin of the left subclavian artery.

Similarly to spinal cord ischemia, the incidence of perioperative stroke has been reported to be lower (1%) in patients who underwent endovascular repair when compared to patients undergoing open surgical repair (4.5%) [49]. These rates are, however, comparable to what has been observed after the treatment of assorted thoracic aortic pathologies [50].

## 8. Long-Term Outcomes

In patients who survive hospital discharge, mid and long-term outcomes after both open and endovascular repair of BTAIs are satisfying [11], with overall reported survival rates nearly 87% at 1 year, 82% at 5 years, and 75% at 10 years [51].

Cheng et al. [52] reported better survival in patients treated with endovascular repair if compared to open surgery (88.9% versus 71.9% at 1 year, 88.9% versus 68.2% at 3 years, and 88.9% versus 65.1% at five years, respectively).

In addition, among new-generation endografts, the Conformable GORE TAG (CTAG) thoracic stent graft (W. L. Gore & Associates, Flagstaff, AZ, USA) has proven to be a safe, effective, and durable option for the treatment of patients with BTAIs, with a reported survival rate of 89% at 5 years [53].

Recently, Gennai et al. performed a systematic review and meta-analysis of long-term reintervention following thoracic endovascular repair for BTAIs. They reported an overall late survival of 95.6% up to 60 months, and they found that aortic reinterventions were rarely required and tended to occur within the first and after the fifth year from the initial procedure [54].

Furthermore, endovascular repair of BTAI seems to not present the sex-based outcome disparities observed after thoracic aortic aneurysm repair, as demonstrated by Rastogi et al., who found no significant association between sex and perioperative outcomes or long-term mortality after TEVAR for BTAIs. The authors explained this contrast by differences in the pathology, demographics, and anatomic factors in these patients [55].

However, the benefit of endovascular repair over open surgical repair in the perioperative period may be counteracted by its unknown long-term effect in a predominantly younger population.

One of the main concerns, in fact, is about the need for long-term graft surveillance, which is mandatory given the fact that following endograft placement for BTAI, the “healthy diameter” of the aorta undergoes remodeling and increases, the more the amount of endograft oversizing [56]. Furthermore, it is also unknown how the endograft will respond to physiologic aortic aging.

Thoracic aortic surveillance is usually performed with chest contrast-enhanced CT, which may pose a challenge for both radiation exposure and contrast-induced nephropathy.

The optimal follow-up program is still not defined and may vary by the institution [39].

Long-term data about outcomes of blunt aortic injuries managed nonoperatively are limited.

## 9. Conclusions

Blunt thoracic aortic injury is a life-threatening problem that requires a high index of suspicion based on the mechanism of injury and a prompt diagnosis in trauma centers with appropriate facilities. In people who survive the traumatic event, endovascular treatment has become the mainstay of treatment whenever feasible, with lower perioperative results if compared to open surgery and promising long-term outcomes. Concerns remain about the need for long-term surveillance after endovascular grafting.

## Figures and Tables

**Table 1:** Description of the most important features of the Gore C-TAG^®^ and the Medtronic Valiant Captivia™, according to Instruction for Use.

Device	Sheath Access Diameter	Proximal Aortic Reference Diameter	Proximal Neck Length Required	Distal Neck Length Required	Available Measures	Tapered Configuration Available
Gore C-TAG^®^	18–24 F	16–42 mm	≥20 mm	≥20 mm	Ø: 21–45 mm Length: 100–200 mm	yes
Medtronic Valiant Captivia™	22–25 F	18–42 mm	≥15 mm	≥15 mm	Ø: 22–46 mm Length: 107–212 mm	yes

## Data Availability

Not applicable.
